# A Newer and Better Era

**Published:** 1900-03

**Authors:** 


### A NEWER AND BETTER ERA.

THE signs of the times are many of an awakening all over our country to the need of better veterinary sanitary police measures. Now that some seventeen States have recognized the peril to their live-stock industries in admitting within their borders dairy or breeding stock from sister States where bovine tuberculosis prevails, and demand at the border lines certificates of health showing a freedom from tuberculosis, there dates a period when this source of perpetuation of the " white man's " plague shall be under control and surveillance, and with thorough dairy inspection for every city and town there will be a lessening in the death-rate of children that can only be faintly estimated to-day. Every State in the Union should take similar steps in protecting its livestock owners, and the people who consume the products of the dairy should fully sustain those charged with official authority and lawmaking in placing every safeguard around these mutual interests from such now recognized and known dangers.

